# Diamine Fungal Inducers of Secondary Metabolism: 1,3-Diaminopropane and Spermidine Trigger Enzymes Involved in β-Alanine and Pantothenic Acid Biosynthesis, Precursors of Phosphopantetheine in the Activation of Multidomain Enzymes

**DOI:** 10.3390/antibiotics13090826

**Published:** 2024-09-01

**Authors:** Juan Francisco Martín, Paloma Liras

**Affiliations:** Departamento de Biología Molecular, Área de Microbiología, Universidad de León, 24071 León, Spain; paloma.liras@unileon.es

**Keywords:** β-alanine, pantothenic acid, polyamines, putrescine, spermidine, spermine, 1,3-diaminopropane, filamentous fungi, phosphopantetheinyl modification, modular synthetases, secondary metabolites

## Abstract

The biosynthesis of antibiotics and other secondary metabolites (also named special metabolites) is regulated by multiple regulatory networks and cascades that act by binding transcriptional factors to the promoter regions of different biosynthetic gene clusters. The binding affinity of transcriptional factors is frequently modulated by their interaction with specific ligand molecules. In the last decades, it was found that the biosynthesis of penicillin is induced by two different molecules, 1,3-diaminopropane and spermidine, but not by putrescine (1,4-diaminobutane) or spermine. 1,3-diaminopropane and spermidine induce the expression of penicillin biosynthetic genes in *Penicillium chrysogenum*. Proteomic studies clearly identified two different proteins that respond to the addition to cultures of these inducers and are involved in β-alanine and pantothenic acid biosynthesis. These compounds are intermediates in the biosynthesis of phosphopantetheine that is required for the activation of non-ribosomal peptide synthetases, polyketide synthases, and fatty acid synthases. These large-size multidomain enzymes are inactive in the “apo” form and are activated by covalent addition of the phosphopantetheine prosthetic group by phosphopantetheinyl transferases. Both 1,3-diaminopropane and spermidine have a similar effect on the biosynthesis of cephalosporin by *Acremonium chrysogenum* and lovastatin by *Aspergillus terreus*, suggesting that this is a common regulatory mechanism in the biosynthesis of bioactive secondary metabolites/natural products.

## 1. Introduction: The Search for Inducers of the Biosynthesis of Secondary Metabolites in Fungi

Filamentous fungi are able to produce an impressive array of secondary metabolites with very diverse chemical structures and biosynthetic pathways. The biosynthesis of particular classes of secondary metabolites respond to specific inducers in combination with transcriptional factors, sometimes associated with other regulatory proteins. This is the case with methionine, which enhances the expression of genes for cephalosporin biosynthesis in *Acremonium chrysogenum* [1,2,3,4]. The induction by methionine of cephalosporin is associated with the formation of arthrospores [5,6] mediated by the central component Axl2 of the bud site selection system [7]. Similarly, the regulation of penicillin in *Penicillium chrysogenum* (reclassified as *Penicillium rubens*) is well known to be exerted: (1) by glucose through the CreA transcriptional regulator [8] combined with a putative ligand proposed to be a hexose-phosphate [9,10] and (2) by the forkhead regulators FKH1 and PcRFX1 [7,11,12,13], although the ligand molecules that bind to these regulatory factors are still unknown. The same occurs in other fungal secondary metabolite biosynthetic systems, e.g., the LovE regulator of *Aspergillus terreus* that controls the expression of genes for lovastatin biosynthesis is induced by diamines [14,15,16]. In *Aspergillus flavus*, aflatoxin formation is controlled by the regulatory protein AflR [17]. In addition, spermidine induces the formation of sclerotia and aflatoxin biosynthesis [18].

In our search for inducers of the expression of penicillin biosynthesis genes, we looked for extracellular signalling molecules in the spend culture broths of *P. chrysogenum* and *A. chrysogenum*. An autoinducer molecule was isolated from spend broth cultures and was identified by MS and NMR in both fungi as 1,3-diaminopropane (1,3-DAP) [19]. A second inducer in the β-lactam-producing fungi was identified as the triamine spermidine. Addition of 1,3-DAP to the cultures increases expression of the penicillin biosynthesis genes *pcbAB*, *pcbC* and *penD*, and prolonged the life span of penicillin gene transcripts in the producing cells [20]. Surprisingly, the induction effect is exerted by 1,3-DAP and spermidine but not by 1,4 diaminobutane (putrescine) or spermine [19]. Both 1,3-DAP and spermidine also have a stimulatory effect on the expression of lovastatin biosynthetic genes that results in an increase in the production of this anticholesterolemic agent in *A. terreus* and in a three days advance of the maximal production level in relation to non-supplemented cultures [21]. Also, the biosynthesis of cephalosporin is stimulated by the addition of 1,3-DAP or spermidine to the culture medium [22,23]. Using a high-cephalosporin-yield strain the production increases by 15–20%; this results in accumulation of the intermediate deacetylcephalosporin C but not its conversion to cephalosporin C, indicating that these inducers affect specifically early and middle enzymes in the cephalosporin biosynthetic pathway [23].

**Figure 1:**
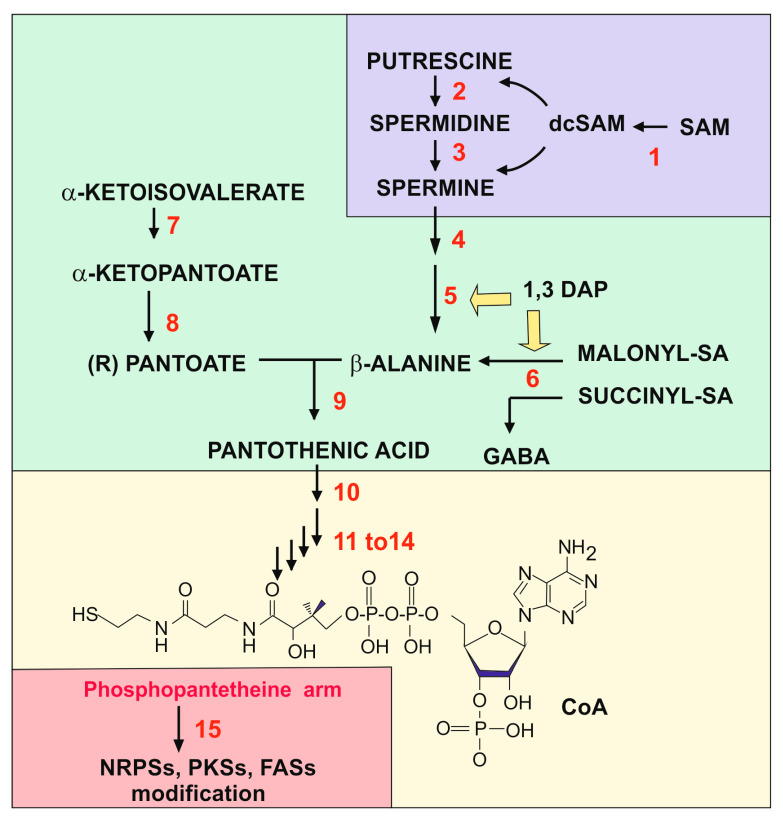
Biosynthetic pathway that links all the compounds studied in this review. The figure has been divided into colored fragments that correspond to the pathways of polyamines (section II, purple color), the pathway for pantothenic acid (section III, green color), the pathway for CoA (section III-2, yellow color), and the modifications by phosphopantetheine (section IV, pink color). The structure of polyamines, β-alanine, panthotenic acid, and dcSAM can be found in other figures of the article. The enzymes, labelled in red, are the following: (1) SAM decarboxylase; (2) spermidine synthase; (3) spermine synthase; (4) polyamine oxidase, FMS1; (5) 3-aminopropanal aldehydedehydrogenase; (6) β-alanine aminotransferase; (7) α-ketopantoato hydroxymethyl-transferase; (8) dehydropantoate reductase; (9) β-alanine-pantoate ligase; (10) pantothenate kinase (Cab1 or PanK); (11–14) enzymes named in yeasts Cab2 to Cab4: phosphopantothenoylcysteine, a metabolite formed by the deamination of valine by the branched chain amino acid aminotransferase phosphopantothenoylcysteine synthetase, phosphopantothenoylcysteine decarboxylase, phosphopantetheine adenylyl transferase, and dephospho-CoA kinase; (15) phosphopantetheinyl transferase. Note that 1,3-DAP is an inducer of the 3-aminopropanal aldehyde dehydrogenase and the semialdehyde aminotransferase.

Proteomic studies of the response of *P. chrysogenum* metabolism to the 1,3-DAP inducer reveal that this compound produces a rearrangement of the metabolic networks in this filamentous fungus, with particular relevance to enzymes involved in the biosynthesis of β-alanine and pantothenic acid [24]. 1,3-DAP is not considered to be one of the major diamines in fungi [25] but is in the slime mold *Dictyostelium discoideum*, in which the intracellular concentration of diamines was determined; 1,3-DAP is present at a 5 mM concentration while other diamines are in the range of 1.6 mM for spermidine and 10 mM for putrescine [26]. The biosynthetic origin of 1,3-DAP and its secretion in fungi is still poorly known. In plants, it is known to be formed by the cleavage of spermine by a polyamine oxidase. In yeasts, 1,3-DAP has been reported to arise by a rare cleavage of N-acetylspermidine by the Fms1 polyamine oxidase [27], and it has been proposed that 1,3-DAP exerts a regulatory role on the metabolism of spermidine and spermine [26]. We review in this article the available information on the biosynthesis of β-alanine, pantothenic acid, and coenzyme A (CoA) in different yeasts and filamentous fungi (Figure 1), and the effect of these molecules, induced by 1,3-DAP and spermidine, on the phosphopantetheinylation of enzymes involved in the biosynthesis of secondary metabolites and fatty acids.

## 2. Polyamines in Fungi and the Origin of the Propylamine Unit for the Biosynthesis of β-Alanine

Polyamines are organic compounds that contain two, three, or four cationic amino groups. They are ubiquitous and play essential roles for life in all living beings. The three major polyamines are the diamine putrescine, the triamine spermidine, and the tetraamine spermine, all of which occur in most filamentous fungi. Due to their strong polybasic nature, polyamines interact with acid polymers such as DNA, RNA, oligonucleotides, and also some proteins. Polyamines interact with DNA, modifying its configuration, and stabilize RNAs, thus affecting gene expression. Their interaction with proteins affects the stability of many of them and particularly that of some ion transporters. The interaction of polyamines with the translation initiation factor eIF-5A affects the initiation of protein synthesis [28,29]; these interactions are not discussed further in this article since this work is focused on the effect of polyamine-derived inducers on the biosynthesis of bioactive secondary metabolites.

**Figure 2:**
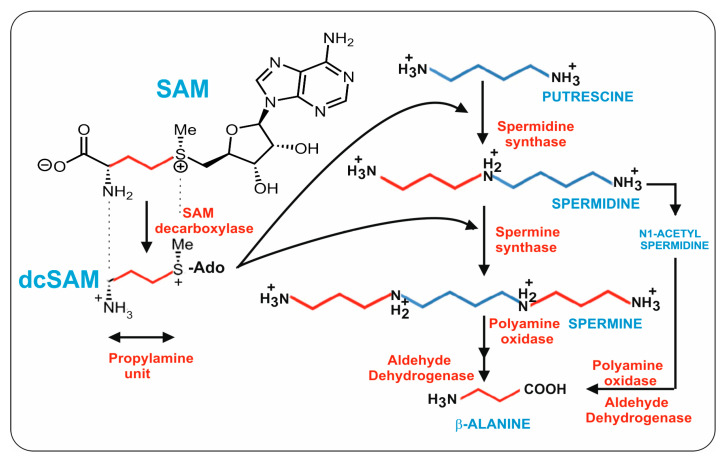
Biosynthetic pathway of spermidine, spermine, and β-alanine. The four carbon units originating from putrescine are indicated in blue. The three carbon propylamine units originating from dcSAM are shown in red. The compound names are shown in blue and the enzyme names are in red.

Putrescine is formed by the decarboxylation of ornithine and is converted into spermidine by fusing with a propylamine unit; an additional propylamine moiety converts spermidine to spermine (Figure 2). An important question from the point of view of β-alanine and pantothenic acid biosynthesis is the origin of the three carbon propylamine units. These propylamine units derive from S-adenosylmethionine (SAM) by the combined action of two enzymes: first, SAM is decarboxylated to decarboxyl-SAM (dcSAM, also named dc-Adomet) by the action of the SAM decarboxylase, and then the three-carbon propylamine unit is transferred from dcSAM to putrescine by the spermidine synthase.

The SAM decarboxylase plays a key role in the splitting of SAM, exposing the three-carbon unit. The reaction mechanism of this enzyme is very complex and has received considerable attention. The SAM decarboxylase encoding gene (named *spe2*) of *N. crassa* was cloned by complementation of a *spe* mutant of this fungus, and in the same work the homologous gene from *Aspergillus nidulans* cDNA was isolated [30]; no introns were detected in the *N. crassa spe2* gene. The *N. crassa* Spe2 protein contains 503 amino acids and has a molecular weight of 54,721 Da. The *A. nidulans spe2* gene partial cDNA sequence encodes 306 amino acids and corresponds to the N-amino terminal end of the enzyme of *N. crassa.* The homologous protein of *P. chrysogenum* (KZN87968) has 59.8% identity to that of *N. crassa* and 67.3% to the protein of *A. nidulans*. The SAM decarboxylases of these fungi and also that of *S. cerevisiae* contain several conserved characteristic sequences that include sites for autocleavage and for interaction with putrescine. The *N. crassa* enzyme contains the sequence ^98^YVLSESSMFV^107^, which is almost identical to the self-processing site of human and yeast SAM decarboxylases. The functionality of *N. crassa* SAM decarboxylase was confirmed by: (1) complementation of a *spe2* mutant, (2) enzymatic reaction in a coupled assay using putrescine as a co-substrate, and (3) by site-directed mutation of conserved sequences that destroy the enzyme activity.

Later, the SAM decarboxylase gene (*sadA*) of the dimorphic fungus *Penicillium marneffei*, a pathogen of immune-compromised persons, was cloned by complementation of a previously isolated mutant of this fungus [31]. The SAM decarboxylase defective mutant grows very poorly in defined minimal medium and is defective in spore germination and conidiation. All these defects were restored by complementation with the cloned *sadA* gene or by addition of spermidine to the medium, indicating that the SAM decarboxylase is essential for spermidine biosynthesis, as in other fungi. The cloned gene encodes a protein of 497 amino acids and is 72% identical to that of *P. chrysogenum*.

**Figure 3:**
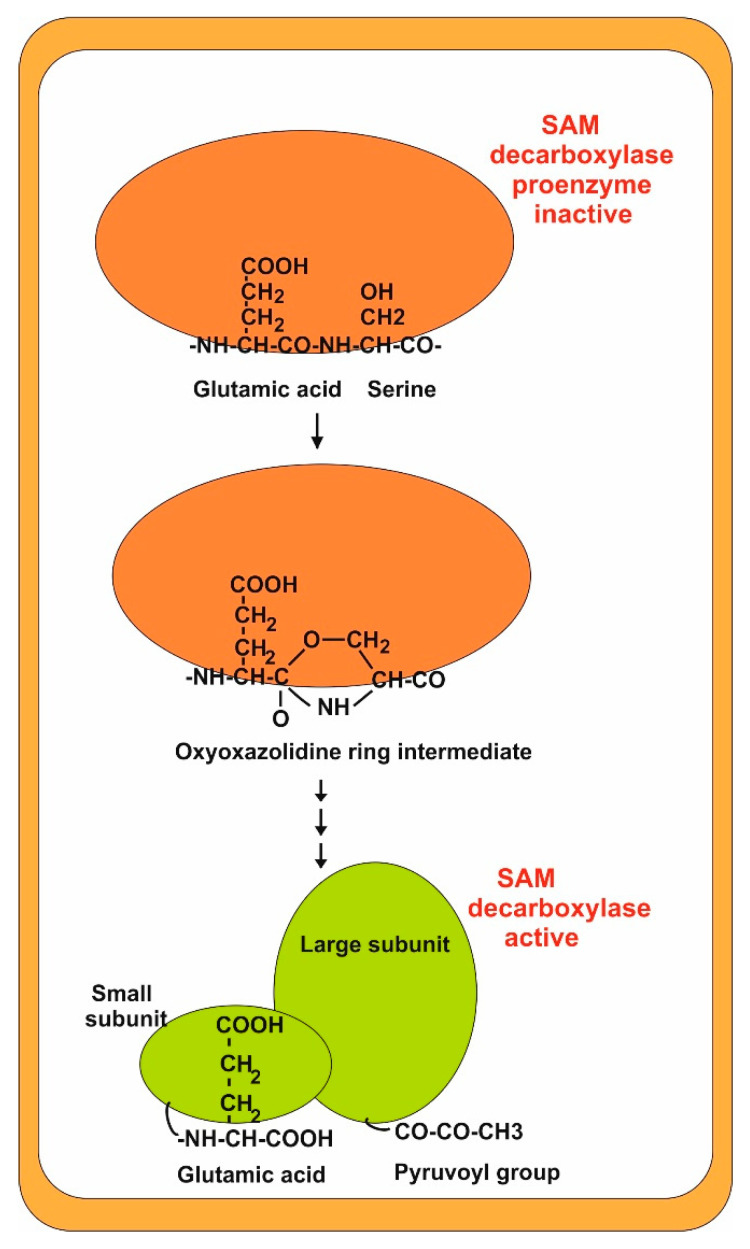
Cleavage process of SAM decarboxylase. The inactive proenzyme is shown in red. The active form (dimer) is shown in green. Note the formation of a pyruvoyl group at the terminus of the large subunit.

Studies in different organisms show that the SAM decarboxylase is synthesized as an inactive proenzyme that undergoes self-cleavage resulting in the active enzyme. SAM decarboxylase cleavage takes place within a glutamyl-serine dipeptide (leucine-serine in *N. crassa* enzyme) in positions 100–101, and the proenzyme produces a dimer of the two resulting subunits α and β (44 and 11 kDa). The amino acid sequence surrounding the cleavage site is well conserved in other fungal SAM decarboxylases, e.g., the cleavage site in *P. chrysogenum* is ^100^ES^101^. Later, the serine residue at the N-terminal position is converted to a pyruvoyl group by deamination [32,33,34]. This pyruvoyl group is involved in the substrate (SAM) decarboxylation reaction [35] (Figure 3). Interestingly, when the pyruvoyl group is transaminated to alanine, the enzyme activity of the modified protein is lost. In summary, the SAM decarboxylase cleavage process in fungi is entirely similar to that in humans, indicating that this mechanism is extremely well conserved in different eukaryotic organisms [33,36]

A putrescine-binding amino acid sequence is present in some decarboxylases [36] and, indeed, binding of putrescine is required for decarboxylase activity, although putrescine is not a proper substrate of the enzyme reaction [30]. In *N. crassa*, binding of putrescine to SAM decarboxylase is needed for enzyme activity, but it is noteworthy that putrescine does not seem to stimulate the cleavage rate [30]; however, in the human enzyme, binding of putrescine stimulates the processing of SAM decarboxylase in contrast to what occurs in *N. crassa.* Crystallization studies have supported that putrescine enters into a deep hole lined by acidic amino acids buried near to the subunit interaction region and the binding of putrescine reorganizes the structure of the protein modulating its enzymatic activity.

A high SAM decarboxylase activity is very important to reach the proper levels of spermidine and other polyamines, but on the other hand it has to be limited because otherwise the SAM levels would be depleted which would impair methylation reactions in the cell; indeed, the decarboxylated product, dcSAM, does not serve as a methyl donor in the methylation reactions. Putrescine modulation of the cleavage process is one of the mechanisms by which SAM decarboxylase activity is controlled [37]. Bioinformatic analysis has shown that *S. cerevisiae* SAM decarboxylase contains 396 amino acids and is smaller than that found in most filamentous fungi enzymes, which are in the range of 500 amino acids [30,38]. This size difference is due to two internal amino acid sequences in the large α subunit of the *N. crassa* enzyme that are missing in *S. cerevisiae* [30].

Since ornithine decarboxylase and SAM decarboxylase catalyze the early step in the biosynthesis of polyamines, they are primary targets for inhibitor design and have been the subject of numerous biochemical and structural investigations [36]; these authors proposed a detailed mechanism of the steps involved in the serinolysis process, including the formation of an oxyoxazolidinic ring intermediate [34,36].

### 2.1. Aminopropyl Transferases: Formation of Spermidine and Spermine

The major polyamines, spermidine and spermine, are formed by two consecutive enzymes, collectively named aminopropyl transferases. Following SAM decarboxylation, the terminal aminopropyl group of dcSAM is transferred to an amino receptor, either putrescine to form spermidine, or spermidine to form spermine (Figure 2) [39,40].

In the model organism *A. nidulans*, disruption of the spermidine synthase leads to spermidine auxotrophs that are defective in cell division, conidiation, and the formation of secondary metabolites [41]. Similar results were observed in spermidine synthase mutants of *A. flavus*, a well-known plant pathogen that affects many plant seeds crops including maize, peanuts, and cotton seeds, where it produces the potent carcinogen aflatoxin [42]. The spermidine synthase mutant does not sporulate, but the wild-type phenotype is restored by complementation with the gene encoding spermidine synthase [18]. It is noteworthy that supplementation of the wild-type strain cultures with spermidine improves the differentiation of *A. flavus* and the production of sclerotia and aflatoxins [18]. Spermidine, in addition to its role as substrate for the formation of spermine, has a key role in the modification of the translation–initiation factor eIF-5A [43]. This modification of the translational initiation factor eI-F5A, common to fungi and in all other eukaryotes, has been studied in detail in *S. cerevisiae* and *Fusarium graminearum* [43,44,45].

Due to its positive charge, the spermidine interacts with DNA in a specific configuration at certain nucleotide sequences and this has an important role in fungal metabolism [46]; however, in some exceptional cases, as in *S. cerevisiae*, disruption of the spermidine synthase encoding gene *spe4* does not affect the growth of *S. cerevisiae*, indicating that it has no essential role in this yeast [39].

### 2.2. Conversion of Spermidine in Spermine

The tetraamine spermine is one of the major polyamines in many living beings. Spermine is present in ascomycetes but has been described to be absent in some other filamentous fungi [25]. Both spermine and spermidine are present in *Aspergillus oryzae* [47], although spermine is absent in *N. crassa* and *Ustilago maydis*, among other fungi [25]. Spermine is synthesized from spermidine by the incorporation of an aminopropyl group to the N1-amino group of spermidine; in other words, spermine contains two aminopropyl carbon units (Figure 2). This reaction is catalyzed by a second aminopropyl transferase (named spermine synthase) that utilizes the aminopropyl group coming from dcSAM. This aminopropyltransferase is similar to the spermidine synthase that introduces the first aminopropyl group from decarboxylated SAM into putrescine. Both enzymes have 35 to 40% identity in *S. cerevisiae*, suggesting that in eukaryotes these enzymes were originated by gene duplication and later evolutive specialization. It is noteworthy that spermine synthase plays an important role in vegetative growth and differentiation in fungi, e.g., it is present in the mycelium, ascospores, and sclerotia of *Sclerotinia sclerotiorum* [48].

### 2.3. Polyamine Oxidases

**Figure 4:**
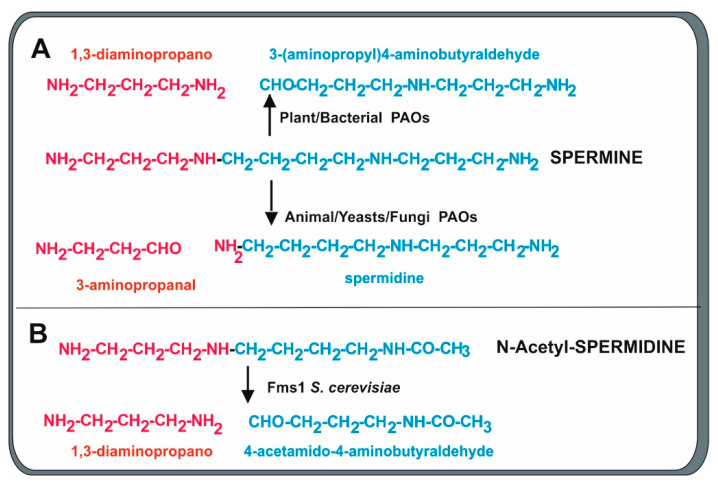
Cleavage mechanisms of oxidation of spermine and N-acetylspermidine by polyamine oxidases of different origins. Red and blue colors indicate the fragment formed from the cleavage. (**A**) Bacterial and plant PAOs produce 1,3-diaminopropane and 3-aminopropyl)-4-aminobutyraldehyde. Animal, yeast, and fungal PAOs preferentially form 3-aminopropanal, a precursor of β-alanine, and spermidine. (**B**) The Fms1 PAO of *S. cerevisiae* also cleaves N-acetylspermidine, forming 1,3-diaminopropane and 4-acetamido-butanal.

An important step in the metabolism of polyamines is the formation of 3-aminopropanal, an immediate precursor of β-alanine from spermine or spermidine by a polyamine oxidase (PAO). Polyamine oxidases in eukaryotes form 3-aminopropanal and spermidine from spermine (Figure 4). The best-known polyamine oxidases are the human N-acetylpolyamine oxidase and spermidine oxidase (SMO), and the yeast polyamine oxidase, encoded by the *fms1* gene [49]. The *S. cerevisiae* Fms1 enzyme was initially reported by Joets et al. [50] to contain 506 amino acids and is 31% similar to the polyamine oxidase of *Candida albicans*, indicating that there are notable differences in the oxidases of different yeasts. Further characterization of this enzyme was made by Landry and Sternglanz [27], who observed that *S. cerevisiae* Fms1 was a flavoprotein with an intense yellow color that contains an FAD prosthetic group in a ratio of 1:1 FAD/enzyme molecule. The first observation of polyamine conversion to β-alanine was based on the finding of mutants in the *fms1* gene that lack biosynthesis of β-alanine and pantothenic acid [51]. The *fms1*-disrupted mutants require β-alanine for growth and the growth was also restored by complementation with a wild-type allele of the *fms1* gene. Oxidative cleavage of spermine forms spermidine and the three-carbon compound 3-aminopropanal that is later oxidized to 3-aminopropionic acid (β-alanine) [27,52]. Substrate specificity studies demonstrate that Fms1 uses spermine but not free spermidine as substrate. In addition, it efficiently splits N1-acetylspermine and N1-acetylspermidine while N8-acetylspermidine is a poorer substrate [27,52]. Free spermidine cannot be oxidized by Fms1, and this is convenient for the metabolism of yeast since it saves spermidine required for the formation of hypusine, a component of the translation initiation factor. In this respect, the yeast Fms1 is similar to the human PAO that is unable to degrade free spermidine.

The crystal structure of yeast Fms1 complexed with spermine has been resolved using the single wavelength anomalous diffraction phasing method. *S. cerevisiae* Fms1 is a dimer in solution and in its crystalline structure and has a molecular weight of 120 kDa [52]. The cofactor FAD binds no-covalently to Fms1, adopting an elongated form. Crystallization studies established that this enzyme contains an FAD binding domain with a Rossmann fold conformation and a spermine binding domain, which is linked via hydrogen bonds in such way that the spermine C11 is close to the catalytic center and to the FAD molecule. This arrangement, which is common to animal cells and yeasts, allows the oxidation at spermine C11, which is close to the FAD binding site, and the enzyme releases spermidine and 3-aminopropanal that can be converted into β-alanine [52]. Importantly, in oxidases in which the spermine C9 carbon (instead of the C11) is close to the FAD site, the enzyme will release 3 (aminopropyl)-4 aminobutyraldehyde and 1,3-diaminopropane as occurs in bacterial and plant PAOs [53,54]. In contrast, mammalian cells contain two separated enzymes: the flavoprotein aminooxidase (PAO) and the spermidine oxidase (SMO), which differ by two or three orders of magnitude in the preference of free spermine [55,56,57]. Additional evidence indicates that these two forms may correspond to alternative splicing of the enzyme mRNA [58].

#### Polyamine Oxidase in *Penicillium chrysogenum*

*Y*ears ago, Kobayashi et al. [59] described a polyamine oxidase in cell cultures of *P. chrysogenum*. The enzyme is extracellular and uses N-acetyl polyamines including N-acetylspermidine and N-acetylspermine as substrates, but not free spermidine; in addition, it also uses spermine releasing spermidine and free aminopropanal. This is consistent with the large amount of 3-aminopropanal required for β-alanine biosynthesis in the high penicillin producing strains. This is the same pattern of substrate specificity as that of the homologous human enzyme; however, at that time the gene encoding this enzyme was not available. A *P. chrysogenum* gene encodes a putative PAO (KZN84195) that has 533 amino acids with a 31% identity to both the *S. cerevisiae* and the human PAO homologous proteins. We have investigated the presence of an FAD binding motif in this *leP. chrysogenum* PAO following the description of the FAD binding domain in *S. cerevisiae* PAO [52] and other FAD-dependent oxidases [60]. The FAD binding motif has a Rossmann fold configuration (β1α1β2α2β3), indicating that the protein belongs to the glutathione reductase subfamily of FAD-dependent enzymes. A comparative search indicates that the FAD binding domain in *P. chrysogenum* extends from amino acid 48 to 85 and includes scattered amino acids that form part of the FAD binding motif [52].

### 2.4. The 1,3-DAP Inducer Triggers Formation of Aldehyde Dehydrogenases That Convert 3-Aminopropanal to β-Alanine

Proteomic studies of 1,3-DAP supplemented cultures of *P. chrysogenum* showed four spots that are isoforms of a protein that corresponds to an aldehyde dehydrogenase which are absent in non-supplemented cultures [24]. Analysis of internal peptides of these isoforms revealed that all of them correspond to protein KAJ6152794 encoded by the gene Pc18g02760 in the genome of *P chrysogenum* Wis 54-1255 [61]. This gene contains four introns and encodes a protein of 502 amino acids. The same *P. chrysogenum* protein with a slightly different number of amino acids is listed in different databases because of the lack of unequivocal definition of the methionine residue at the protein translation initiation site. The *P. chrysogenum* protein has a 50% identity to the *S. cerevisiae* ALD2 protein that was shown to catalyze the conversion of 3-aminopropanal to 3-amino propionic acid (β-alanine) in a pioneering study by White et al. [62]. This finding supports the conclusion that 1,3-DAP triggers the biosynthesis of β-alanine in *P. chrysogenum.*

### 2.5. Other Proteins Related to β-Alanine Biosynthesis in P. chrysogenum

Proteomic studies of 1,3-DAP or spermidine-supplemented *P. chrysogenum* cultures revealed the presence of an additional protein spot that increased 2.75-fold in the presence of 1,3-DAP and 4.4-fold in the presence of spermidine [24]. This spot was absent in spermidine non-supplemented cultures. These two proteins correspond to isoforms of an aldehyde aminotransferase that converts, in the biosynthetic direction, an acid semialdehyde, e.g., succinyl-semialdehyde (succinyl-SA) or malonyl-semialdehyde (malonyl-SA), into the corresponding amino acids. These isoforms are encoded by Pc21g17880 [61], a gene with four introns, which encodes a protein annotated as γ-aminobutyric acid (GABA) aminotransferase. The protein contains 498 amino acids and has an estimated molecular weight of 51.7 KDa. Interestingly, this protein is 80.7% identical to the γ-aminobutyric acid transaminase GatA of *A. nidulans* [63]. In the forward direction, these types of enzymes act on malonyl-SA, converting it to 3-aminopropionic acid (β-alanine), while succinyl-SA is converted into GABA upon transamination. The reverse reaction of the enzyme has been reported using GABA as substrate, which is converted in succinyl-SA that is oxidized to succinate and enters the tricarboxylic acid cycle and increases the potential energy of the cells. Although the catabolic reaction of this aminotransferase has received much more attention than its biosynthetic role, the conversion of malonyl-SA to β-alanine or succinyl-SA to GABA has relevance in the biosynthesis of these metabolites. GABA is a very important neurotransmitter in the cells of animal brains and, therefore, has been intensively studied in this function in the nervous system. In fungi, GABA plays important roles in many aspects of metabolism including cell growth, differentiation, and the production of secondary metabolites, and GABA homeostasis is maintained by a balance between GABA biosynthesis and degradation. In yeasts, the γ-aminobutyric acid aminotransferase (GABA aminotransferase or GatA) has been widely studied because, in the catabolic direction, it is used for the utilization of γ-aminobutyric or β-alanine as nitrogen sources. The GatA protein was first described in *S. cerevisiae* [64] and purified in *Candida guillermondii* by der Garabedian et al. [65]. Cultures grown in ammonia show very little GatA activity, but the enzyme activity increases strongly in the presence of GABA as the sole nitrogen source. This induction by GABA facilitated the purification of the enzyme to almost homogeneity and its kinetic studies. The purified GatA enzyme of *C. guillermondi* is a homodimer of 55kDa that acts as ω-aminotransferase, using 4-aminobutyric acid as the best nitrogen donor, and has low activity on β-alanine. Structural analogues such as α-alanine and 2-aminobutyrate (aspartic acid) are inhibitors of the transamination. The purified GatA contains one molecule of pyridoxal phosphate (PLP) bound to each subunit as occurs in other aminotransferases [65].

#### 2.5.1. Duplicated GABA Aminotransferases in Some Yeasts: Specificity for GABA or for β-Alanine as Substrates

As indicated above, mammals [66] and the yeast *S. cerevisiae* contain a single GABA aminotransferase able to degrade both GABA and β-alanine. However, some other yeasts have been reported to have separate enzymes for the catabolism of GABA and β-alanine. This is the case of *Saccharomyces kluyvery*, which contains two related aminotransferases; one of these, SkUga1, is involved in the transamination of only GABA, whereas the other, SkPYD4, is implicated in the transamination of both GABA and β-alanine, with preference for β-alanine as substrate and using GABA with low activity. Both enzymes have PLP as a cofactor and use α-ketoglutarate, but not pyruvate, as an amino group acceptor [67]. The genes SkUga1 and SkPYD4 were cloned by complementation of different *S. kluyvery* mutants. Phylogenetic analysis of different yeasts revealed that only the genomes of *Candida albicans* and *Debaromyces hansenii* contain two GABA aminotransferase genes, similar to SkUga1 and SkPYD4. The SkPYD4 gene arose, many years ago, by gene duplication of the consensus “Uga1” aminotransferase in an ancestral progenitor of these yeasts, and one of the duplicated genes evolved to a different transaminase, PYD4, with distinct substrate specificity [67]. *S. kluyvery* null mutants defective in both aminotransferases still have a residual growth on GABA, meaning that there is a third, still unknown, aminotransferase that exerts the same function. The PLP-mediated mechanism of transamination is identical to other group II aminotransferases, forming a Schiff base intermediate between PLP and a lysine residue of the protein. This molecular mechanism is supported by the structure of the protein deduced from crystallization studies of the *Escherichia coli* GatA protein [68,69].

#### 2.5.2. GABA Aminotransferases in Filamentous Fungi

In addition to the ω-aminotransferases of several yeasts, there are reports on the cloning and characterization of genes for similar enzymes in different filamentous fungi including *A. nidulans* [63], *Fusarium graminearum* [70], *Neurospora crassa* [71], and the basidiomycete *Ustilago maydis* [72]. We summarize briefly here the characteristics of the aminotransferases of these fungi particularly with respect to the specificity for β-alanine or GABA as substrates.

The GABA aminotransferase (GatA) of *A. nidulans* was cloned during early studies on the nitrogen regulation of different enzymes in this fungus. The *A. nidulans gatA* gene encodes a protein of 498 amino acids and has a deduced molecular mass of 55 kDa [63]. Noteworthy, this size and molecular mass are near identical to those of *P. chrysogenum* aminotransferase induced by 1,3-DAP or spermidine in proteomic studies [24]. The GatA activity of *A. nidulans* is induced by β-alanine and controlled by the regulatory protein AmdR, which also regulates the expression of other relate nitrogen metabolism genes.

In the *F. graminearum* genome, Bönnighausen et al. [70] found two putative GABA aminotransferase genes that encode proteins sharing 55% identity to the *A. nidulans* GatA enzyme. Both genes were disrupted, obtaining the mutants *gat1* and *gat2*. The substrate specificity of knockout mutants *gat1* and *gat2* was tested by supplementation with GABA or β-alanine. It was concluded that the *gat1* gene encodes an aminotransferase with specificity for β-alanine whereas the *gat2* gene encodes an aminotransferase that uses preferentially GABA as substrate. However, both genes were required for the metabolism of GABA since the double mutant was able to grow only when supplemented with GABA [70]. These *F. graminearum* mutants defective in the GABA aminotransferase display stunted growth, are sensitive to oxidative stress, and show defective respiration and oxygen consumption in the mitochondria, which results in lower pathogenicity. In summary, the biosynthetic and catabolic reactions of GABA maintain a balance of this compound that is adequate for growth and pathogenicity.

A functional analysis of the GABA metabolism and the GABA shunt have been recently made in *Neurospora crassa* [71]. This shunt bypasses two reactions in the tricarboxylic acid cycle by converting glutamate (five carbons) to GABA (four carbons), and then the GABA transaminase forms succinic-SA that enters into the TCA cycle. The enzymes involved in this shunt are glutamate decarboxylase, GABA aminotransferase, and succinyl-SA dehydrogenase. Mutants in two of these enzymes, the glutamate decarboxylase and the succinyl-SA dehydrogenase, were unable to grow in β-alanine indicating that β-alanine is a substrate for the GABA aminotransferase in the GABA shunt. However, these mutants still grow on GABA, suggesting that there should be an additional GABA aminotransferase in this fungus.

In addition to the characterization of GABA metabolic enzymes in ascomycetes, the GABA aminotransferase of the basidiomycete *U. maydis* was cloned in early studies [72]. It is noteworthy that the GABA aminotransferase of this fungus is induced by both β-alanine and GABA. Interestingly, a *U. maydis* mutant disrupted in the GABA aminotransferase was still able to grow on β-alanine as the only nitrogen source, supporting the conclusion that there is probably a second GABA aminotransferase in this fungus, although this has not been confirmed.

## 3. From β-Alanine to Phosphopantetheinyl Activation of Multimodular Enzymes

Pantothenic acid is an essential component in the metabolism of all living beings. This compound is synthesized in bacteria, yeasts, filamentous fungi, and plants but not in animals that require it as a vitamin in their diet (vitamin B5). Only a few exceptional examples of yeasts have been described to be natural auxotrophs of pantothenic acid, e.g., some yeasts involved in sake fermentation and the biocontrol yeast *Hanseniaspore meyeri* [73,74]. Furthermore, pantothenic acid in the form of 4′-phosphopantetheine is part of the prosthetic group that modifies fatty acid synthases (FASs) and numerous biosynthetic enzymes including non-ribosomal peptide synthetases (NRPSs) and polyketide synthases (PKSs). Phosphopantetheine is linked by a phosphoester bond between its phosphate group and a conserved serine residue in all acyl- and peptidyl-carrier proteins [75]. An important derivative of phosphopantotenic acid is the coenzyme A (CoA) that is an essential molecule in the metabolism of all living beings, either in its free form or as acetyl-CoA and other acyl-CoA derivatives [76]. These compounds play key roles in anabolic reactions of microbial metabolism (e.g., the formation of fatty acid and a variety of metabolic intermediates) and in catabolic reactions to generate energy (e.g., the catabolism of pyruvate to acetyl-CoA and its oxidation in the tricarboxylic acid cycle, forming NADH and ATP) [77,78]. Furthermore, CoA is the donor of phosphopantetheine to the phosphopantetheinyl transferases, as reported in *A. nidulans*, *P. chrysogenum*, and several other fungi [79,80,81].

### 3.1. Pantothenic Acid in Yeast and Filamentous Fungi

**Figure 5:**
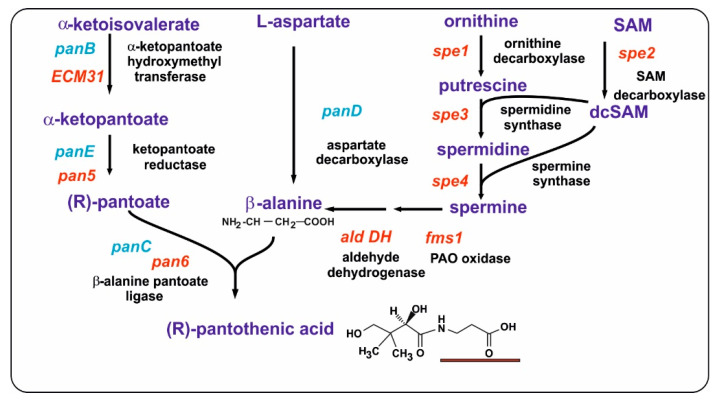
Biosynthesis of pantothenic acid in yeasts and *Escherichia coli*. The biosynthetic pathway leading to pantothenic acid formation is shown. The names of the enzymes are indicated in black letters. The genes in *E. coli* are shown in blue. The genes in yeasts are shown in red. Note that, in *E. coli*, β-alanine is formed directly by the decarboxylation of aspartate (PanD). No *panD* gene/enzyme occurs in yeasts. The β-alanine moiety in pantothenic acid is underlined.

Pantothenic acid biosynthesis is similar in bacteria, yeasts, and filamentous fungi, except for the origin of the β-alanine moiety [51]. In *E. coli*, pantothenic acid is formed by the condensation of pantoate and β-alanine by the pantoate-β–alanine ligase, encoded by *panC*. Four genes are required for pantothenic acid formation in *E. coli* and two enzymes encoded by *panB* and *panE* are needed to form pantoate from α-ketoisovalerate, a metabolite formed by the deamination of valine by the branched chain amino acid aminotransferase. The β-alanine moiety is formed in *E. coli* by the decarboxylation of aspartate by the aspartate decarboxylase, encoded by the *panD* gene (Figure 5).

In fungi, the biosynthesis of pantothenic acid proceeds through several reactions, starting with α-ketoisovalerate. In the first step, there is a transfer of an hydroxymethyl group that converts α-ketoisovalerate to α-ketopantoate via the enzyme α-ketopantoate hydroxymethyltransferase; then, the α-ketopantoate is converted to pantoate by the α-ketopantoate reductase, and finally there is a condensation of pantoate with β-alanine by the β-alanine pantoato ligase (also called pantothenic acid synthetase, PAS) (Figure 5).

In contrast to bacteria, early studies revealed that *S. cerevisiae* requires pantothenic acid for growth that may be replaced by β-alanine; however, later research showed that *S. cerevisiae* is not a strict pantothenic acid auxotroph. It was concluded that *S. cerevisiae* does not form β-alanine by the aspartate decarboxylase and indeed there is no *panD* homologous gene in the genome of *S. cerevisiae* [51,82]. It was then proposed that, in yeast, β-alanine is synthesized by a different pathway involving the diamines putrescine, spermidine, and spermine [51], and this was confirmed later in several studies [27,61]. Further research showed that yeasts form β-alanine, required for pantothenic acid production, via polyamine metabolism, synthesized by four enzymes encoded by the *spe* genes and completed by the FAD-dependent polyamine oxidase encoded by *fms1* [61].

Following the discovery of the pantothenic acid pathway in *E coli* and other enterobacteria, the genes involved in pantothenic acid biosynthesis in yeast and *A. nidulans* were reported [83,84]. However, surprisingly little information is available on the molecular characteristics of the genes and proteins of this biosynthesis pathway in fungi. In *A. nidulans*, a gene encoding the α-ketopantoate-hydroxymethyl transferase was cloned by th complementation of mutants blocked at this stage in pantothenic acid biosynthesis [83], restoring its ability to grow in the absence of pantothenic acid. This enzyme forms α-ketopantoate from α-ketoisovaletare (Figure 5). The cloned gene contains a 60 bp intron and encodes a protein (named ECM31) of 349 amino acids with a predicted molecular weight of 37.7 KDa. This protein sequence has 38% identity and 48% overall similarity to the protein encoded by the *panB* gene of *E. coli*. The *A. nidulans* PanB protein contains two well-conserved sequences, one of them ^103^LVGDS^107^ near the N-terminal region and the other, ^263^GIGAG^267^, in the middle of the protein, which are conserved in all prokaryotic and eukaryotic α-ketopantoate-hydroxymethyl transferases [83]. Interestingly, the mutant used as a receptor strain in the cloning process was shown to contain a mutation in the G^267^ residue that belongs to the second conserved sequence. Heterologous expression of this gene in *E. coli* resulted in high α-ketopantoate hydroxymethyltransferase activity, confirming the identity of the enzyme encoded by the cloned *A. nidulans* gene [83].

Phylogenetic studies of pantothenic acid biosynthetic genes in archaea, bacteria, fungi, and some animals established that the pantothenic acid biosynthetic pathway was assembled separately in archaea and in bacteria, although the overall pathway is similar using the same precursors. Eukaryotic organisms obtain pantothenic acid biosynthetic genes from an ancestral progenitor of bacteria [76]. In order to clarify the conservation of the pantothenic acid biosynthetic genes in different yeasts and filamentous fungi, we made comparative alignments using first the *E. coli* PanB, PanE, PanC, and PanD proteins as probes (Supplementary Table S1A) and then the ECM31, Pan5, and Pan6 proteins of *A. nidulans* as probes (Supplementary Table S1B). In the comparative studies using *E. coli* proteins as probes, we observed an interesting finding: namely, there is a high identity conservation between the PanB and PanC proteins from *E. coli* and those of the fission yeast *Schizosaccharomyces pombe* (68 and 59%, respectively), whereas the identity with the corresponding gene in the budding yeast *S. cerevisiae* is lower (36 and 38%). This lower identity also occurs with several filamentous fungi, including the model fungi *A. nidulans* and *P. chrysogenum* (36 to 39% identity). Importantly, none of the yeasts or filamentous fungi showed in their genomes any gene encoding a protein homologous to PanD (aspartate decarboxylase) that in *E. coli* forms β-alanine; this confirms the early finding of PanD absence in *S. cerevisiae* [51]. In all studied yeasts and filamentous fungi, the conservation of the PanE enzyme (dehydropantoate reductase) is always lower than that of the other proteins of the pathway (hydroxymethyltransferase and pantoate–β-alanine ligase), especially in *S. cerevisiae* and *Sc. pombe.* These findings suggest that dehydropantoate reductase evolved differently from other pantothenate biosynthetic enzymes.

When the comparison in different yeast and fungi was made with the *A. nidulas* proteins as probes, the overall conservation was higher since the probe was of fungal origin and in general the conservation of PanB and PanC was also higher than that of PanE. There was a higher identity between the *A. nidulans* and the *A. fumigatus* proteins and lower identity with proteins of more distantly related fungi such as *P. chrysogenum* (order Eurotiales), *A. chrysogenum* (order Hypocreales), and *N. crassa* (order Sordariales).

#### 3.1.1. The α-Ketopantoate Reductase: An Enigmatic Enzyme

This enzyme forms pantoate using dehydropantoate as a substrate (Figure 5). Several *S. cerevisiae* mutants defective in the dehydropantoate reductase (Pan5) were tested to know their effect on the biosynthesis of pantothenic acid [85]. A *S. cerevisiae* mutant in the *pan5* gene that corresponds to *E. coli panE* (22.4–40% amino acid identity/similarity) was still able to grow in the absence of pantothenic acid although to a lesser extent than the parental strain, suggesting that there is an alternative gene encoding this reductase. An isoenzyme encoded by a different *pan5*-like gene was found in the genome of *S. cerevisiae* (21.4–33% amino acid identity/similarity to the *E. coli* homologue). This second gene was disrupted, and the mutant was still able to grow at a lower rate than the parental strain. Even the double mutant (in *pan5* and *pan5*-like genes) was not strictly a pantothenic acid auxotroph, indicating that another dehydropantoate reductase activity is likely to be encoded by a different reductase gene not closely related to that of *E. coli panE* [85].

#### 3.1.2. The Pantoate and β-Alanine Condensing Enzyme

Pantothenic acid is formed by the condensation of ATP-activated pantoate and β-alanine. The condensation is catalyzed by pantothenic acid synthetase (PAS), encoded in *E. coli* by *panC*. The homologous *pan6* gene of *S. cerevisiae* was cloned together with the genes for the same enzyme in *Lotus japonicus* and *Oryza sativa* (rice) [84]. The *S. cerevisiae pan6* gene was able to complement an *E. coli* mutant defective in the homologous gene and *pan6* was also similar to the cloned genes of lotus and rice. These three pantothenic acid synthetases were similar throughout the entire amino acid sequence, but the *S. cerevisiae* is longer at the amino terminal end (36 additional amino acids in relation to the *E. coli* enzyme). The *S. cerevisiae* Pan6 sequence was 46% identical to that of *E. coli.* The *S. cerevisiae* PAS N-terminal extension contains an amino acids sequence similar to that of plants’ mitochondrial transport motifs, which suggests that the *S. cerevisiae* enzyme might be located in mitochondria, although this hypothesis needs to be experimentally confirmed [84].

The cloned *S. cerevisiae pan6* gene, encoding PAS, contains 309 amino acids and was expressed in *E. coli* as well as those of lotus and rice and the enzymes were purified. The purified proteins showed PAS activity and used preferentially the free lineal pantothenic acid form as a substrate rather than its cyclic pantoyl lactone form.

#### 3.1.3. Intercellular Cross Feeding of Pantothenic Acid

An important point is the use of external pantothenic acid produced by other organisms in the vicinity of fungal colonies for the growth of pantothenic acid auxotrophic strains. In addition to the genes involved in pantothenic acid biosynthesis, *S. cerevisiae* contains a pantothenic acid transporter (Fen2) that is able to scavenge pantothenic acid from the surrounding medium [86]. Mutants defective in the *pan6* gene cannot grow in the absence of pantothenic acid, but growth is recovered when the culture is supplemented with pantothenic acid indicating that the compound enters the cell [74]. Interestingly, some yeasts with small genomes, e. g. *Hanseniaspora* (telemorph of *Kloeckera*) *meyeri* strains lack the genes *ECM3* and *pan6* (equivalent to *E coli panB* and *panC*), but are fully able to grow when supplemented with pantothenic acid. A bioinformatic analysis showed that the *H. meyeri* genome has up to six genes for pantothenic acid transport, similar to the *fen2* gene of *S. cerevisiae.* One of these genes was able to complement a *S. cerevisiae pan6* mutant that grows poorly when supplemented with pantothenic acid; the *H. meyeri* complementing pantothenic acid transporter is a Major Facilitator Superfamily (MSF) protein with 12 α-helixes and topological studies of the sequence indicate that it is located in the cell membrane [74]. These results point to a significant communication between different organisms mediated by pantothenic acid in various habitats in nature.

### 3.2. Formation of Coenzyme A from Pantothenic Acid

In order to synthesize CoA from pantothenic acid, this molecule has to be combined with cysteine, forming an intermediate that is later decarboxylated and finally combined with ATP to form the CoA molecule. The pathway has been studied in mammals and the homologous genes and reactions are well known in *E. coli* and *S. cerevisiae*, showing that this pathway seems to be similar in all living beings. In the first of these reactions, pantothenic acid is phosphorylated with ATP to form 4′-phosphopantothenate, a reaction catalyzed by the panthotenate kinase (PanK) (Figure 1), which is the limiting reaction in the whole pathway. The reaction catalyzed by PanK is a key regulatory point, since this enzyme in mammals is feedback-inhibited by CoA, acetyl-CoA, and to a lesser extent other CoA derivatives, e.g., succinyl-CoA, although in *E. coli* is only regulated by CoA. The next reaction of the pathway is performed by the phosphopanthotenoylcysteine synthetase (PPCS) that links 4′-phosphopanthotenate to cysteine. The phosphopantothenate–cysteine intermediate is then decarboxylated to form 4′-phosphopantetheine by the 4′-phosphopantothenoylcysteine decarboxylase (PPCDC). Incorporation of ATP to 4′-phosphopantetheine gives rise to dephospho-CoA and finally the dephospho-CoA kinase (DPCK) releases Coenzyme A [87].

#### 3.2.1. The Pantothenate Kinase

The first step in the conversion of pantothenic acid to CoA is catalyzed by the pantotenate kinase (*panK* or *cab1*) (Figure 1). The *panK* gene of *A. nidulans* was cloned by Calder et al. [88] using a cDNA library to complement a temperature-sensitive *E. coli panK* mutant. The *A. nidulans panK* gene has three introns and encodes a protein of 420 amino acids and a molecular weight of 46.9 kDa. The protein has 44.8% identity and 60.2% similarity to *S. cerevisiae* PanK, which contains 367 amino acids [88]. The *A. nidulans panK* gene was expressed efficiently in *E. coli* and the enzyme activity that converts pantothenate to phosphopantothenate was measured in vitro. The pure enzyme requires only Mg^2+^ ions but no other cofactors, and ATP may be substituted by GTP although with lower efficiency. A comparative study of *A. nidulans* and *E. coli* PanK proteins revealed that both enzymes are inhibited by acetyl-CoA and free CoA, but they differ in the degree of inhibition by these two compounds; in contrast to the *E. coli* enzyme that was more sensitive to free CoA, the *A. nidulans* enzyme was strongly inhibited by acetyl-CoA but poorly inhibited by CoA. In summary, the regulation by acetyl-CoA and the much lower effect of CoA derivatives on *A. nidulans* PanK has a significant effect on the conversion of pantothenic acid to phosphopantothenic acid and avoids an intracellular excess of pantotenate; when intracellular panthotenate is in excess, it is excreted in bacteria and fungi [74].

#### 3.2.2. Characterization of the Saccharomyces Cerevisiae PanK Gene

An entirely different strategy was used to clone the pantothenate kinase of yeasts. In S. *cerevisiae*, this gene was cloned using a mutant deficient in fatty acid biosynthesis that, however, contained a complete set of genes for fatty acid biosynthesis but lacked phosphopantetheine formation; the mutant was able to grow at 30 °C but failed to grow at 37 °C. A S. *cerevisiae* DNA fragment that complemented the S. *cerevisiae* sensitivity to 37 °C contained a gene similar to that of *A. nidulans* and other eukaryotic *panK* genes [85]. To confirm that the fragment contained the *panK* gene of S. *cerevisiae*, the authors obtained in the wild-type strain a knockout mutant in the putative *panK* gene and found that, indeed, the null strain was also temperature sensitive. The similarity of the S. *cerevisiae* pantothenate kinase gene was higher with eukaryotic homologous genes than with the *E. coli* gene. It is noteworthy that both the *E. coli* and the murine *panK* genes complemented the S. *cerevisiae* knockout mutant, thus confirming that the cloned gene, which was named *cab1* for CoA biosynthesis, was a *panK* homologous. A precise analysis in the temperature-sensitive strain identified a missense mutation G^351^S, in a position that was conserved in all the pantothenate kinases. The PanK activity of the mutant was assayed and was found to indeed be temperature sensitive. The structure of the deduced *S*. *cerevisiae* pantothenate kinase is consistent with the crystal structure of the *E. coli* enzyme [89].

A bioinformatic analysis of S. *cerevisiae* genome identified putative genes for the next enzymes of the CoA biosynthesis pathway (named *cab2* to *cab5*). Null mutant alleles of each of these genes were obtained in heterozygous diploids, but after their sporulation no haploid carrying the mutation could be obtained, indicating that the genes were essential. Confirmation of the function of these genes was obtained after heterologous complementation of the heterozygous diploid carrying the mutation with the homologous genes of *E. coli* (*coaBC*, *coaD*, *coaE*).

## 4. Activation of Antibiotic Biosynthetic Enzymes by Phosphopantetheinylation

Many antibiotics and other secondary metabolites’ biosynthetic enzymes are activated by modification with a phosphopantetheinyl group, a reaction that is catalyzed by phosphopantetheinyl transferases (PPTases) [90,91]. These enzymes include non-ribosomal peptide synthetases, polyketide synthetases, fatty acid synthases, and hybrid PKS-NRPS synthetases (Figure 1). Some enzymes involved in primary metabolism, as the α-aminoadipate reductase involved in the biosynthesis of penicillins [92,93,94], are also subject of this type of modification. Importantly, in contrast to other protein modifications, such as phosphorylations or acetylations [75], the phosphopantetheynil addition is essential for the activation of these enzymes from the “apo” inactive form to the “holo” active modified form. Phosphopantetheinylation of NRPSs, PKSs, and FASs proceeds by establishing a covalent bond between the phosphate group of the phosphopantetheine and a conserved serine in the acyl carrier protein of PKSs and FASs or the peptidyl carrier proteins of NRPSs [95]. There are thousands of PPTases in the databases of bacterial, fungal, and plant genomes, but only a few of them have been characterized in detail, particularly in actinobacteria and in filamentous fungi [75,96]. Only in one database are there more than 700 entries for PPTases [97]. A bioinformatic analysis of *A. nidulans* genome revealed that it contains 27 genes for PKSs and 14 genes for NRPSs [98] and that, in the genome of *P. chrysogenum*, 20 PKSs, 10 NRPSs, and 2 hybrid NRPS-PKS have been annotated [61]; all these enzymes require PPTase modification for the adequate production of the bioactive secondary metabolites.

### How Many PPTases Are Involved in the Activation of Multidomain Synthetases?

PPTases are classified into three types according to their substrate specificity: Class I includes the low molecular weight PPTases (122 to about 200 amino acids), with a narrow substrate spectrum [90,99]. The class II PPTases activate eukaryotic FASs, forming a domain of these large enzyme complexes [100]. Class III contains PPTases with wider substrate specificities designated Sfp-type (for surfactin synthetase) PPTases, which are about twice the size of the enzymes of type I and are frequent in the modification of bacterial and fungal NRPSs and PKSs [90,101,102]. Three PPTases have been found in *S. cerevisiae*. One of them is involved in the activation of fatty acid synthases and is integrated in the large type I FAS complex [100]; a second PPTase is a mitochondrial enzyme that activates prokaryotic type acyl-carrier proteins. In addition, in yeasts a third PPTase encoded by the *lys5* gene was required for activation of the α-aminoadipate reductase encoded by the *lys2* gene [103]. In fungi, the best-known PPTases are those of *A. nidulans* and *P. chrysogenum* [79,80,81]. The first PPTase cloned in fungi was that of *A. nidulans* using a mutant deficient in pigmentation as receptor [79,80]. The PPTase-defective mutant failed to form pigments and was unable to synthesize penicillin and a siderophore, which suggests that penicillin, the cellular pigment, and the siderophore are synthesized by multidomain enzymes that require activation by phophopantetheinylation. These results suggest that a unique PPTase is responsible for the three effects. However, recent evidence indicates there are at least three PPTases in the yeasts and filamentous fungi studied. Further research of the role of the PPTase gene both in *A. nidulans* and *P. chrysogenum* indicated that there are other PPTase genes for the biosynthesis of specific secondary metabolites. Márquez Fernández and coworkers [80] observed that the CfwA/NpgA gene of *A nidulans* was required for sporulation and for the synthesis of several polyketides but not for fatty acid biosynthesis. Among the polyketide-derived metabolites these authors identified in extracts from *A. nidulans* cultures shamixanthone, emericellin, and dehydroaustinol, although the exact PKSs that are activated by the PPTase were not elucidated. Ferrichrome type of siderofores in *A. nidulans* have intermediate peptides in their biosynthesis, in particular a peptide synthesized from the precursors N^5^-Acetyl-N^5^-hydroxyornithine, serine, and glycine [104,105]; this ferrichrocin peptide precursor is formed by a NRPS that requires a PPTase for its activity.

The PPTase gene of *P. chrysogenum* was cloned from a library of the wild-type strain *P. chrysogenum* NRRL1951 and later this sequence was confirmed in the genome of *P. chrysogenum* Wis54-1255, a derivative of the wild-type strain [61,81]. The *P. chrysogenum* PPT1 sequence has 412 amino acids and is similar to that of *A. nidulans* and other ascomycetes. *P. chrysogenum* requires a high PPTase activity, particularly in the very high penicillin producing strains. However, there is only one copy of the PPTase gene, e.g., this gene is not located in the 56.8 kb region that is amplified in tandem in the high penicillin producing strains [106,107]. A *P. chrysogenum ppt* mutant is unable to produce penicillin or pigments but can still synthesize other secondary metabolites such as roquefortine. This suggests that this dipeptide-like metabolite does not involve modification by PPT1 of a PKS or a NRPS in its biosynthesis [108,109]. Roquefortine is synthesized by the condensation of histidine with dimethylallytryptophan by a cyclodipeptide synthetase, forming a diketopiperazine ring by an enzyme which does not seem to require PPT1, although we cannot exclude that there is a different PPTase that may modify this enzyme. An example of the important role of the PPTases in the biosynthesis of secondary metabolites is that amplification of the copy number of the *P. chrysogenum* PPT1 gene expressed from its own promoter increases significantly (30) the production of penicillin [81].

The PPTase gene (*ppt*) of *P. chrysogenum* and *A. chrysogenum* is required for the biosynthesis of penicillin and cephalosporin because is essential for the activity of the peptide forming α-aminoadipyl-cysteinyl-valine (ACV) synthetase, a NRPS, but the knockout of *ppt* does not affect the synthesis of fatty acids, indicating that they are formed by a fatty acid synthase that does not use this PPTase [81].

Investigation of the α-aminoadipate reductase in *P. chrysogenum* [92,110] indicated clearly that this enzyme is essential for lysine biosynthesis in this fungus and also for penicillin production because α-aminoadipate is an essential precursor of β-lactam antibiotic biosynthesis; the enzyme was characterized and it was observed that Lys2 is similar to a monomodular NRPS that recognizes adipic acid and, in addition, it contains a reductase domain in the C-terminal region [93,94]. In conclusion, two enzymes of the penicillin pathway require phosphopantetheinylation, including the α-aminoadipate reductase and the ACV synthetase. Complementation of the *ppt* mutant and biosynthetic studies of penicillin demostrated that the PPTase1 of *P. crhysogenum* was able to carry both proteins activation but it does not affect fatty acid biosynthesis; this gene was sufficient to activate the proteins involved in penicillin biosynthesis as is also the case of *A. nidulans* [79].

## 5. Conclusions and Future Outlook

The major polyamines putrescine, spermidine, and spermine affect numerous cellular metabolic reactions and the biosynthesis of hundreds of microbial products. However, the role of minor polyamines such as 1,3-DAP has received less attention, even though several reports describe an important regulatory effect on the biosynthesis of different antibiotics and other bioactive secondary metabolites, including penicillin, cephalosporin, and lovastatin, among others [21,22,24]. Most available information has focused on the molecular mechanism of action of the major polyamines on animal cells, particularly in human tissues [28,29,45]; however, information about the mechanisms by which 1,3-DAP induces the expression of enzymes for different secondary metabolites in producer fungi is still limited. It has been demonstrated that 1,3-DAP increases the expression of penicillin biosynthetic genes in *P. chrysogenum* and also those for lovastatin biosynthesis in *A. terreus* [20,21]. Transcriptional studies and proteomic analysis of the response of *P. chrysogenum* and other fungi to the addition of 1,3-DAP or spermidine show that these agents produce an important rearrangement of cell metabolism, including both (1) primary metabolism resulting in the availability of precursors for secondary metabolites and (2) most important in the expression of their gene clusters and activation of enzymes for the biosynthesis of those metabolites [24]. In addition, an important finding was the observation that 1,3-DAP enhances the life span of penicillin gene transcripts [20] and this is in agreement with the report that spermidine increases the longevity of yeasts and also of some animal cells [111,112]. In addition to the role of inducers in fungi, the biosynthesis of some bacterial metabolites, e.g., cephamycin C in *Nocardia lactamdurans*, is enhanced by addition of 1,3-DAP to the cultures; this results in a 3- to 6-fold increase of the production of this antibiotic. This is due to the overexpression of genes for cephamycin biosynthesis such as the early genes *lat*, *pcbAB*, and *pcd*, encoding lysine 6 aminotransferase, ACV synthetase, and piperidein-6-carboxylate dehydrogenase, respectively. Also, the enzymes involved in the late methoxylation steps in cephamycin biosynthesis were induced after 1,3-DAP addition as shown by immunoblotting assays [113]. In *P. chrysogenum*, proteomic studies showed that 1,3-DAP induces two proteins that increase several folds; these proteins catalyze reactions involved in the biosynthesis of β-alanine from 3-aminopropanal. This finding strongly suggests that β-alanine and pantothenic acid exert a key role on the response of fungi to the inducers. The biological activity of NRPSs, PKSs, and FASs involves phosphopantetheinyl modification of the inactive “apo” forms of these multimodular enzymes. Several secondary metabolite synthetases are activated in fungi by the main PPTase, Ppt1, and therefore it was suggested that a unique PPTase may be responsible for modification of most of the multidomain antibiotic synthetases in *A. nidulans* [79,80]. However, studies in *P. chrysogenum* and *A. nidulans* indicate clearly that at least formation of the fatty acids and sterols is not affected in mutants defective in *ppt1*. It is now clear that there are at least three PPTases in filamentous fungi, one of which is involved directly in the activation of fatty acid synthases and another is present in mitochondria. However, other PPTases that have not been detected so far may occur. In actinobacteria, particularly in *Streptomyces tsukubaensis*, it is known that there are five PPTases with different substrate specificities [96,114]. Further research is needed on this point in filamentous fungi, taking into account the large amounts and diversity of fungal species.

A remaining open question is how the inducers accumulated in the extracellular culture broth during the fermentation transmits signals to the antibiotic-producing cells. 1,3-DAP is not degraded by the putrescine oxidase, or by the diamine:α-ketoglutarate aminotransferase of *S. cerevisiae*, suggesting that the role of this inducer is transmitted by a signal cascade that does not involves its oxidation [115]. However, there are no data on the molecular signalling mechanisms in filamentous fungi; in plants, 1,3-DAP is not oxidized by barley cells, although it seems to be utilized as a nutrient by maize shoots. This subject requires further research to clarify this point. The described advances on the induction of activation of multimodular NRPSs and PKSs by small molecules has great relevance in future applications on the biosynthesis of novel fungal secondary metabolites. It is well established that the biosynthesis of bioactive metabolites responds to changes in the major polyamines in the cell [18], but there is too little information on the role of specific polyamine-derived small diamines such as 1,3-DAP. So far, the effect of this inducer has been reported in *P. chrysogenum*, *A. chrysogenum*, and *A. terreus*, stimulating, at the transcriptional level, the biosynthesis of enzymes involved in the formation of penicillin, cephalosporin, and lovastatin. Taking into account the thousands of fungal species [116] and the large number of secondary metabolites produced by each of them, those studies will provide important information for the overproduction of some of these pharmaceutically important metabolites. Further characterization of additional PPTases will help to enhance the production of secondary metabolites which are silent or poorly expressed. This will provide novel antibiotics, antitumor agents, or other natural products for use in medicine or agriculture. The main difficulty in finding and characterizing alternative PPTase genes in filamentous fungi is that the conservation of the overall amino acid sequences is very low and this limits the use of comparative bioinformatic searches; the elucidation of further PPTases would require confirmation of the function of the putative alternative candidate genes implying gene disruption and complementation. Those studies will open the way for biochemical engineering procedures to elucidate the activation of NRPSs and PKSs by the direct mutation of genes encoding those multidomain enzymes.

## Supplementary Materials

The following supporting information can be downloaded at: https://www.mdpi.com/article/10.3390/antibiotics13090826/s1, Table S1: Identity (%) between the sequence of proteins of the pantothenic acid pathway in different organisms. (A) identity of the proteins in yeast and fungi with the homologous proteins in *E. coli*. (B) identity of the proteins in *Aspergillus nidulans* with the homologous proteins of other filamentous fungi.

## Abbreviations

ATP	adenosine triphosphate
CoA	coenzyme A
1,3-DAP	1,3-diaminopropane
DPCK	dephospho-CoA kinase
FAD	flavin adenine dinucleotide
FAS	fatty acid synthase
GABA	γ-aminobutyric acid
malonyl-SA	malonyl semialdehyde
NADH	nicotinamide adenine dinucleotide reduced form
NRPS	non-ribosomal peptide synthetase
PAO	polyamine oxidase
PAS	pantothenic acid synthetase
PKS	polyketide synthetase
PLP	pyridoxal phosphate
PPTase	phosphopantetheine transferase
PPCS	phosphopanthotenoylcysteine synthetase
PPCDC	4′-phosphopantothenoylcysteine decarboxylase
succinyl-SA	succinyl semialdehyde
SAM	S-adenosylmethionine
dcSAM	decarboxylated S-adenosylmethionine
SMO	spermidine oxidase
