# Supplementary material for: Diamine Fungal Inducers of Secondary Metabolism: 1,3-Diaminopropane and Spermidine Trigger Enzymes Involved in β-Alanine and Pantothenic Acid Biosynthesis, Precursors of Phosphopantetheine in the Activation of Multidomain Enzymes
Source: Antibiotics (Basel). 2024 Sep 1;13(9):826. doi: 10.3390/antibiotics13090826 (PMC11428646; doi:10.3390/antibiotics13090826)
Supplement: Supplementary file 1 [file antibiotics-13-00826-s001.zip › antibiotics-3153445-supplementary.pdf]

Supplementary Material

Table S1. Identity (%) between the sequence of proteins of the pantothenic acid pathway in different organisms. **(A)** identity of the proteins in yeast and fungi with the homologous proteins in *E. coli*. (**B**) identity of the proteins in *Aspergillus nidulans* with the homologous proteins of other filamentous fungi.

	E. coli	***Sc. pombe***	***S. cerevisiae***	***A. nidulans***	***P. chrysogenum***
**(A)**	*panB*	68.3% NP_592822	36.25% CAI1424886: *ECM3*	38.5% AAD37248	39.1% KZN85028
	panE	26.6% Q9HDU6	25.3% AJP39160: *pan5*	32.09% XP_050467642	31.1% KZN86928
	panC	59.6% NP_592821	38.6% CAI4359024:*pan6*	38.9% XP_657809	36.47% KZN88320
	panD	ND	ND	ND	ND
	A. nidulans	***A. fumigatus***	***N. crassa***	***P. chrysogenum***	***A. chrysogenum***
**(B)**	*panB*	83.2% XP_750805	72.6% XP_957955	69.7% KZN850228	70.5% KFH41205
	panE	64.5% XP_754967	28.6% XP_011393943	55.3% KZN86928	31.6% KFH41648
	panC	81.3% KAF4256678	57.90% XP_961962	71% KZN88320	57.2 KFH43534

ND: gene no detectable.
